# Efficacy and safety of acupuncture for postoperative gastroparesis syndrome: a systematic review and meta-analysis

**DOI:** 10.3389/fmed.2024.1494693

**Published:** 2025-01-06

**Authors:** Yichuan Xv, Yiyi Feng, Jiang Lin

**Affiliations:** ^1^Department of Gastroenterology, Longhua Hospital, Shanghai University of Traditional Chinese Medicine, Shanghai, China; ^2^Department of Traditional Chinese Medicine, Sir Run Run Shaw Hospital, School of Medicine, Zhejiang University, Hangzhou, China

**Keywords:** acupuncture, postoperative gastroparesis syndrome, systematic review, acupoint, meta-analysis

## Abstract

**Background:**

Postoperative gastroparesis syndrome (PGS) is a common postoperative complication characterized by epigastralgia, nausea, and vomiting. Acupuncture is widely used to aid recovery, but its efficacy and safety have not been systematically evaluated.

**Method:**

We retrieved randomized controlled trials (RCTs) using acupuncture as the primary intervention from six databases. After study selection and data extraction, a meta-analysis was performed using Review Manager 5.4.1. Study quality was assessed with the Cochrane risk of bias tool, and publication bias was quantitatively evaluated using Egger’s test and was corrected using the trimming and filling method.

**Results:**

A total of 12 RCTs involving 709 participants (363 in the acupuncture group and 346 in the control group) were included. The meta-analysis showed a significantly higher overall response rate in the acupuncture group than the control group [RD = 0.16, 95% CI (0.11, 0.21), *p* < 0.001]. Acupuncture also reduced gastric tube indwelling time [MD = −2.36, 95% CI (−3.14, −1.59), *p* < 0.001], decreased gastric juice drainage [MD = −166.88, 95% CI (−176.57, −156.18), *p* < 0.001], and improved serum motilin levels [MD = 41.65, 95% CI (30.14, 53.15), *p* < 0.001]. Four studies reported no adverse events in either group, but the majority of studies did not provide safety data.

**Conclusion:**

Acupuncture may alleviate clinical symptoms and shorten hospitalization, potentially by enhancing gastrointestinal motility. However, the lack of safety data in the majority of studies raises concerns about the reliability of these findings and the transferability of the results. Future trials should focus on rigorous randomization, blinding, and comprehensive safety reporting to improve the quality of evidence in this field.

**Systematic review registration:**

ID: INPLASY202320035 URL: https://inplasy.com/inplasy-2023-2-0035/

## 1 Introduction

Postoperative gastroparesis syndrome (PGS), a gastrointestinal motility disorder, is a common complication after surgery, characterized by delayed gastric emptying without mechanical obstruction (1). The most common symptoms are epigastralgia, nausea, and vomiting. The main manifestations observed in gastroscopy include gastric fluid retention, weakened gastric peristalsis, anastomotic edema, and chronic inflammation, with no difficulty passing through the anastomotic stoma (2). PGS occurs frequently after upper abdominal surgery, particularly gastric and pancreatic surgeries, but it can also occur after lower abdominal or thoracic surgeries, leading to a prolonged course with distressing symptoms (3). Pharmacological therapy for PGS is aimed at reducing symptoms. Prokinetics and antiemetics are the preferred options, typically combined with standard medical care, which includes gastrointestinal decompression, fasting, and gastric lavage with hypertonic saline (4). Patients need to undergo a course of treatment for at least several weeks before gastrointestinal function recovers. During this period, patients often suffer from anxiety and depression caused by the disease, which significantly deteriorates their quality of life (5).

Acupuncture, an internationally practiced complementary therapeutic method that typically involves the manual insertion of fine needles into specific acupoints, has gained significant attention in recent years. It has a superior effect on regulating gastrointestinal function and is recognized as an ideal treatment option for both organic and functional gastrointestinal diseases (6). Substantial studies have found that it can significantly relieve digestive symptoms such as nausea, vomiting, and abdominal pain (7, 8). Thus, acupuncture is frequently used to relieve symptoms of PGS and shorten the duration of treatment in clinical practice (9).

A systematic review published in 2014 compared acupuncture with usual care/medication (10), and suggested the positive effect of acupuncture therapy in PGS. However, the degree of improvement in symptoms was the only evaluated outcome in the article, lacking the evaluation of objective and biochemical indices. Moreover, the body of research on acupuncture for PGS has increased substantially in recent years, making an updated meta-analysis essential for current clinical practice. Therefore, we retrieved relevant randomized controlled trials (RCTs) and conducted this meta-analysis to systematically evaluate the clinical efficacy and safety of acupuncture in treating PGS.

## 2 Method

We conducted this review in accordance with the Preferred Reporting Items for Systematic Reviews and Meta-Analyses (PRISMA) checklist. We registered the protocol for our review on INPLASY (11). The registration number is INPLASY202320035. The protocol is publicly available on its website.^1^

### 2.1 Inclusion criteria

1. Types of studies: Only randomized controlled trials (RCTs) evaluating the clinical outcomes of acupuncture for PGS were included, regardless of blinding. The language was restricted to Chinese and English.
2. Research subjects: We included patients who met the clinical diagnosis of PGS, regardless of age, sex, nationality, or surgical procedure. However, patients had to be comparable at baseline.
3. Interventions: The control group received conventional treatment (e.g., gastrointestinal decompression, fasting, and application of prokinetic drugs), while the experimental group received additional acupuncture therapy alongside conventional treatment. The acupuncture method applied in our study was defined as the penetration of skin or muscle by needles. Several new acupuncture techniques, such as warm acupuncture, electroacupuncture, and acupoint injection, were also included. These supplementary techniques, based on needle penetration, are believed to enhance the therapeutic effects of acupuncture points. It is worth noting that for studies involving acupoint injection, the same drug should also be administered to the control group, either orally or intravenously. However, studies that used interventions not involving needle penetration, such as earball or moxibustion, were excluded. Studies using a combination of acupuncture and oral herbal decoction were also excluded because additional oral herbal intake would bias our evaluation of acupuncture’s efficacy to varying degrees.
4. Outcomes: The primary outcome was the overall response rate, assessed through improvements in symptoms such as nausea, vomiting, abdominal pain, or distension. The secondary outcomes were: (a) gastric juice draining amount, (b) gastric tube indwelling time, and (c) serum motilin level. Finally, safety data from all included studies were collected to demonstrate the safety of acupuncture therapy in PGS.

### 2.2 Exclusion criteria

The following studies were excluded: (a) duplicate literature; (b) literature with incomplete data or studies where the full text could not be retrieved; and (c) abstracts, conference papers, dissertations, and other non-peer-reviewed documents.

### 2.3 Search strategy

We searched both English and Chinese databases for relevant literature. The English databases searched included PubMed, Embase, and the Cochrane Central Registry of Controlled Trials, while the Chinese databases included the China National Knowledge Infrastructure (CNKI), VIP Database, and Wanfang Data. The search spanned from the inception of each database to March 2024. Search terms used across all databases included: “post-operative,” “gastroparesis,” and “acupuncture.” The search strategy was slightly adjusted to accommodate the differences between English and Chinese databases. The detailed search strategy is shown in Supplementary Table S1.

To ensure comprehensive coverage, a second search was conducted in July 2024, after the initial review was completed, to account for any newly published studies during the review period. However, no new publications meeting the inclusion criteria were identified in the second search.

### 2.4 Selection and data extraction

All retrieved literature was imported into Endnote X9 for subsequent analysis. Duplicate records were removed, and two independent reviewers (YX and YF) screened the literature based on the predefined inclusion and exclusion criteria. Initially, titles and abstracts were assessed to exclude studies that were not RCTs, did not involve acupuncture interventions, or did not focus on PGS. Full-text articles were then reviewed to confirm eligibility, and the reasons for exclusion were recorded.

In cases of disagreement between the two reviewers, a third reviewer (JL) was consulted to resolve the issue. Data extraction was independently performed by the same two reviewers (YX and YF) using a pre-designed extraction form. The following data were collected: study title, first author, publication year, sample size, demographic characteristics, type of acupuncture, duration and frequency of treatment, acupoints used, outcome measures, adverse effects, and any information relevant to the risk of bias assessment.

### 2.5 Assessment of the risk of bias

The methodological quality of RCTs was assessed using the Cochrane Collaboration’s risk of bias tool (12). The following biases were assessed: selection bias, performance bias, detection bias, attrition bias, reporting bias, and other biases. For each item, a rating of “high,” “unclear,” or “low” was assigned.

The two independent reviewers (YX and YF) conducted the assessment. Any disagreements were resolved through discussion until a consensus was reached. In cases where the randomization procedure or other methodological details were unclear, attempts were made to contact the first or corresponding author via email or letter for further clarification. Studies that met the inclusion criteria were retained for subsequent meta-analysis.

### 2.6 Statistical analysis

Meta-analysis of the collected data was conducted using Review Manager software (version 5.4). Dichotomous data were reported as absolute risk difference (RD) using the Mantel–Haenszel method, while continuous data were presented as mean difference (MD). All results were expressed with a 95% confidence interval (CI). Heterogeneity across studies was assessed using the *I*^2^ statistic. An *I*^2^ value of less than 50% indicated no significant statistical heterogeneity, and a fixed-effect model was applied. An *I*^2^ value greater than 50% suggested the presence of statistical heterogeneity, which was further explored using subgroup analysis or sensitivity analysis. If the source of heterogeneity could not be identified and the heterogeneity remained substantial, a random-effect model was used for the combined analysis. In cases where the heterogeneity was too high for quantitative synthesis, a descriptive review was provided, and no further statistical analysis was conducted.

### 2.7 Publication bias assessment

Publication bias was assessed using funnel plots and Egger’s regression test. The funnel plots were used to visually inspect for asymmetry, which may suggest potential publication bias, particularly for outcome indicators with more than 10 studies. Asymmetry in the funnel plot was considered indicative of bias. To quantitatively evaluate funnel plot asymmetry, we performed Egger’s regression test. A *p*-value of less than 0.05 was considered indicative of significant publication bias. In cases of identified publication bias, a trimming method was applied to adjust for errors that might arise from the inclusion of small-scale studies.

## 3 Result

### 3.1 Study selection

**Figure 1:**
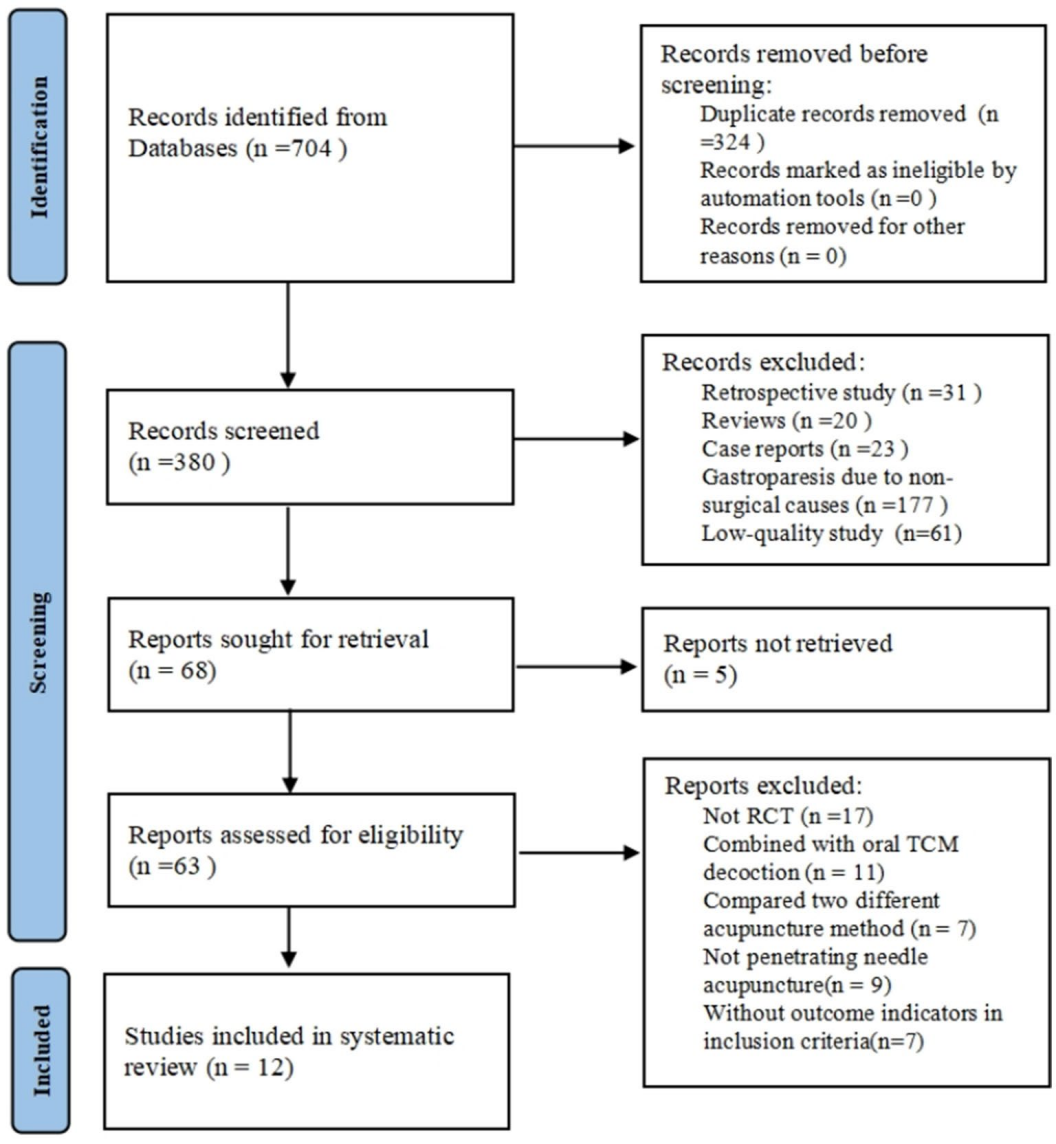
PRISMA flowchart for literature search and screening.

A total of 704 articles were initially retrieved, including 620 from Chinese databases and 84 from English databases. After an initial screening of titles and abstracts, 636 articles were excluded due to duplication (*n* = 324) or irrelevance (*n* = 312). Irrelevant studies included retrospective studies, reviews, case reports, and articles focusing on gastroparesis caused by non-surgical factors. Sixty-eight articles proceeded to full-text evaluation, and 56 studies were excluded after further screening. Ultimately, 12 RCTs were included in the meta-analysis (13–24). The selection process is illustrated in the PRISMA flowchart (Figure 1).

### 3.2 General characteristics and quality of the selected studies

A total of 12 RCTs, comprising 709 participants (363 in the acupuncture group and 346 in the control group), were included. These studies were published between 2013 and 2022. The sample sizes ranged from 36 to 106 participants, with an average age range of 46.25 to 63 years. Regarding acupuncture protocols, ST36 was the most commonly used acupoint, appearing in all studies. Other frequently used acupoints included PC6 and RN25 (used in eight studies), followed by RN6 and SPT25. In all studies, “Deqi” (the sensation of needle arrival, characterized by soreness, distention, and heaviness at the acupoint) was pursued. Needle retention time ranged from 15 to 60 min, with 30 min being the most common duration. Treatment duration varied from 7 to 21 days, with the majority of studies administering acupuncture once a day. The characteristics of the included studies are summarized in Table 1.

**Table 1:** Characteristics of included studies and patients.

References	Patients	Age	Sex (M/F)	Operation form	Study course	Experimental measures	Acupuncture therapy details			Control measures	Adverse events	Outcomes
							Treatment points	Frequency	Duration of each treatment			
Er (13)	I: 22 C: 22	I: 55 C: 55	NR	Abdominal operation	14 days	Electro-acupuncture	RN11, ST36, PC6, RN4, RN10	Once a day	20 min	Usual care and medication	NR	① Gastric juice draining amount ② Efficacy
Lu and Wei (14)	I: 43 C: 43	I: 63 C: 63	I: 25/18 C: 26/17	Thoracic and abdominal operation	10 days	Electro-acupuncture and acupoint injection	PC6, ST36, SP6, LR3	Once a day	30 min	Usual care, medication, and washing the stomach with hypertonic normal saline twice per day	None	① Gastric juice draining amount ② Efficacy
Lan and Sun (15)	I: 20 C: 20	I: 56.82 C: 55.12	I: 7/13 C: 8/12	Abdominal operation	7 days	Electro-acupuncture	RN12, ST36, PC6, GB8	Once a day	30 min	Usual care and medication	NR	① Gastric juice draining amount ② Efficacy ③ Recovery time of gastrointestinal function ④ Gastric tube indwelling time ⑤ Hospital stay
Jin (16)	I: 30 C: 30	I: 60.02 C: 58.6	NR	NR	14 days	Electro-acupuncture	RN12, ST25, SP15, ST36, PC6, SP4, ST37	Once a day	30 min	Usual care	NR	① Gastric juice draining amount ② Efficacy ③ Recovery time of gastrointestinal function ④ Gastric tube indwelling time
Meng et al. (17)	I: 24 C: 12	I: 56 C: 55	I: 14/10 C: 8/4	Abdominal operation	14 days	Electro-acupuncture	PC6, RN12, SPT25, ST36, SP4	Once a day	20 min	Usual care and medication	NR	① Gastric juice draining amount ② Efficacy
Zhang et al. (18)	I: 34 C: 34	I: 46.78 C: 46.25	I: 0/34 C: 0/34	Gynecological operation	7 days	Manual acupuncture	RN12, ST25, LR13, RN4, BL60, BL21, ST36, ST44, ST43	Once a day	25 min	Usual care and oral mosapride (5 mg, 3 times a day)	NR	① Efficacy ② Recovery time of gastrointestinal function ③ Gastric tube indwelling time ④ Hospital stay ⑤ Serum motilin level ⑥ Electrogastrogram
Ouyang et al. (19)	I: 23 C: 22	I: 61.2 C: 60.8	I: 16/7 C: 14/8	Abdominal operation	14 days	Manual acupuncture	RN12, ST36, SPT25, ST37, RN6, PC6, SP4	Every other day	40 min	Usual care and medication	NR	① Gastric juice draining amount ② Efficacy
Dong et al. (20)	I: 31 C: 31	I: 59.12 C: 60.03	I: 13/18 C: 15/16	Abdominal operation	14 days	Electro-acupuncture and warm acupuncture	ST36, SP6, RN6, RN12, ST37	2 times/day	60 min	Usual care metoclopramide (10 mg, intramuscularly, 2 times/day) and washing stomach with hypertonic normal saline	NR	① Serum motilin level ② Efficacy ③ Gastric tube indwelling time ④ Recovery time of gastrointestinal function
Shi et al. (21)	I: 30 C: 31	I: 60.27 C: 59.54	I: 13/17 C: 16/15	Abdominal operation	14 days	Manual acupuncture and acupoint injection	RN13, RN12, RN10, RN6, ST25, PC6, ST36	Once a day	30 min	Usual care, metoclopramide (10 mg, intramuscularly, once a day)	None	① Gastric juice draining amount ② Recovery time of gastrointestinal function
Chen et al. (22)	I: 25 C: 25	I: 60 C: 58	I: 15/10 C: 14/11	Thoracic and abdominal operation	14 days	Electro-acupuncture and warm acupuncture	ST36, RN12, RN6, ST37, SP6	2 times/day	60 min	Usual care, medication and washing stomach with hypertonic normal saline 3 times/day	NR	① Serum motilin level ② Symptom scores ③ Efficacy ④ Recovery time of gastrointestinal function
Yang et al. (23)	I: 55 C: 51	I: 61.53 C: 61.27	I: 26/29 C: 34/17	Abdominal operation	21 days	Manual acupuncture and herbal plaster	PC6, ST36, SP25, RN6, RN8	Once a day	20 min	Usual care, metoclopramide (10 mg, intramuscularly, 2 times/day)	None	① Gastric juice draining amount ② Efficacy ③ Gastric tube indwelling time ④ Symptom scores
Song et al. (24)	I: 27 C: 27	I: 57.24 C: 58.23	I: 16/11 C: 15/12	Abdominal operation	20 days	Acupoint injection	ST36	Once a day	/	Usual care, medication, and washing stomach with hypertonic normal saline 2 times/day	None	① Gastric juice draining amount ② Efficacy ③ Recovery time of gastrointestinal function.

C, control group; I, intervention group; NR, not recorded; PC, pericardium meridian of hand Jueyin; RN, Ren meridians; SP, spleen meridian of foot Taiyin; ST, stomach meridian of foot Yangming; LI, large intestine meridian of hand Yangming; LR, liver meridian of foot Queyin; GB, gallbladder meridian of foot Shaoyang; BL, bladder meridian of foot Taiyang.

### 3.3 Risk of bias in included trials

**Figure 2:**
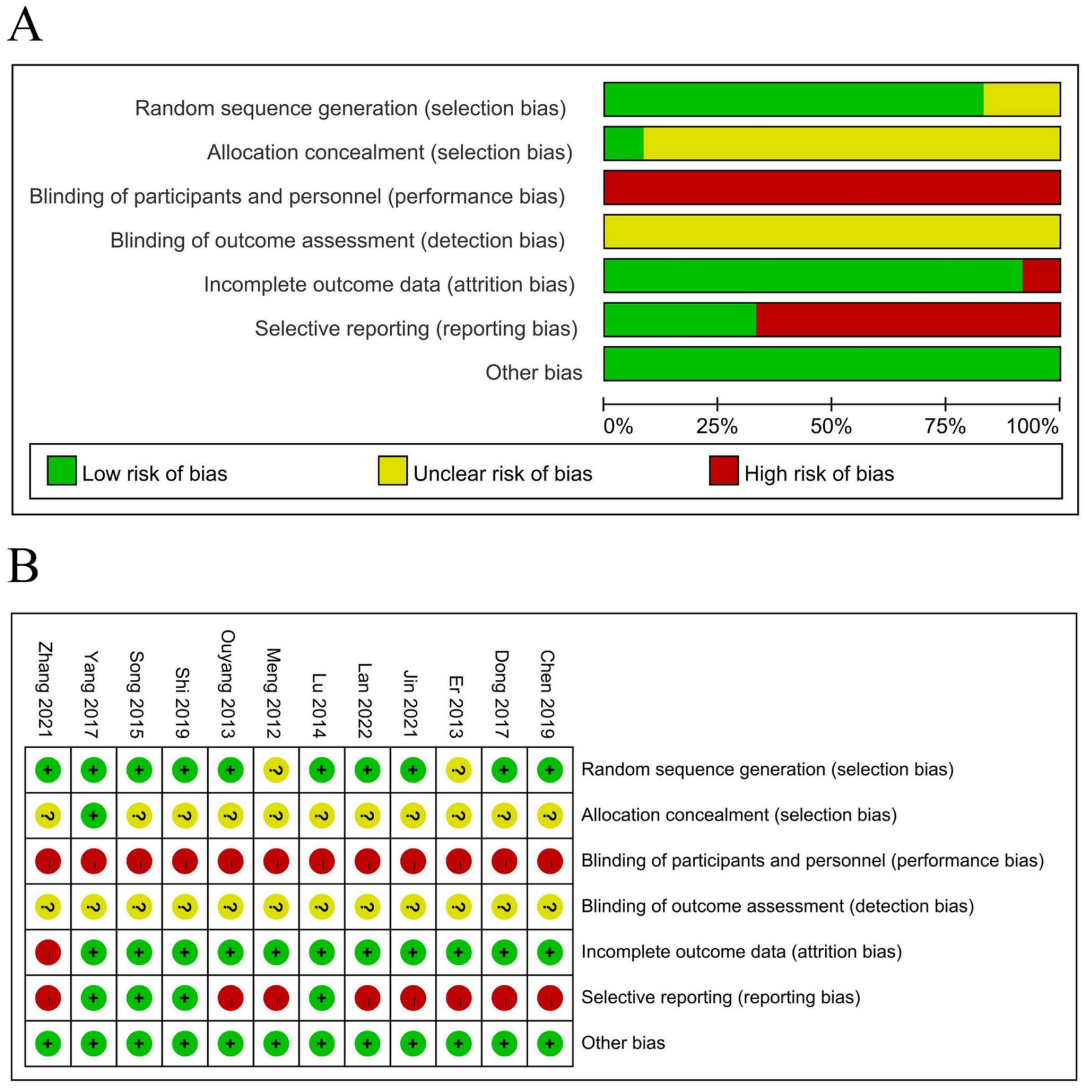
Results of the risk of bias assessment. **(A)** Risk of bias graph and **(B)** risk of bias summary. Red represents high risk, green represents low risk, and yellow represents uncertain risk.

Ten studies (14–16, 18–24) used a random number table for randomization, which was considered to present a low risk of bias. However, two studies were assessed as having an unclear risk of bias due to insufficient information on the randomization method. It is important to note that while the use of a random number table is generally considered a low-risk method, the potential for artificial bias in the process should be taken into account, and the interpretation of these results should be approached with caution. Allocation concealment, achieved using opaque envelopes, was reported in one study (23), which was classified as having a low risk of bias. For the remaining studies, allocation concealment was not explicitly mentioned, and thus, they were assessed as having an unclear risk of bias. None of the studies reported blinding of participants or personnel. As acupuncture was an additional treatment in the experimental group, and the control group did not receive sham acupuncture, all studies were considered to have a high risk of bias in this domain. Blinding of outcome assessment was not mentioned in any of the studies, leading to an unclear risk of bias in this area. Additionally, one study reported the number and reasons for participant dropout (18), which was classified as a high risk of bias. No issues related to missing data were noted in the other studies included in the analysis. Four studies (14, 21, 23, 24) reported the safety data and were assessed as having a low risk of selective reporting. The other studies that did not mention the safety of acupuncture therapy were considered to have a high risk of selective reporting. A summary of the risk of bias assessment is shown in Figure 2.

### 3.4 Results of meta-analysis

#### 3.4.1 The overall response rate of acupuncture therapy

**Figure 3:**
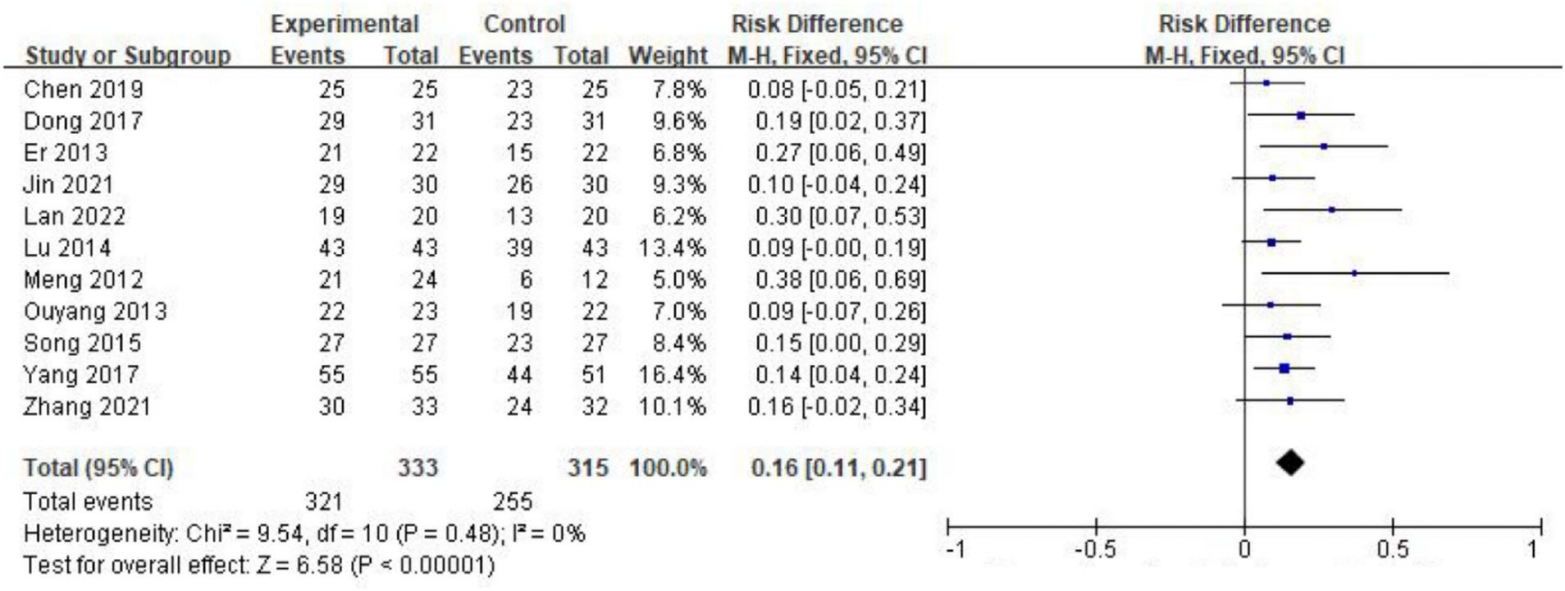
Forest plot of overall response rate after treatment. The coordinate values corresponding to the squares represent the response rate difference between the experimental group and control group of each study, and the range of values spanned by the horizontal lines represents the confidence interval of each study. The diamond at the bottom represents a pooled effect.

The criteria for assessing clinical efficacy in the included studies primarily followed the consensus on the diagnosis and treatment of gastroparesis in China. This consensus outlined clinical symptoms such as nausea, vomiting, abdominal distension, abdominal pain, and gastric drainage for evaluating treatment effectiveness. Eleven studies (13–20, 22–24) involving 648 patients reported the overall response rate after treatment. One study (13) classified improvement into two categories: “recovered” and “ineffective,” while the remaining 10 studies used three categories: “recovered,” “effective,” and “ineffective.” In a subsequent meta-analysis, we combined “recovered” and “effective” as “responsive” to create dichotomous data. In the study by Shi et al. (21), the time to recovery of gastrointestinal function was used as the primary outcome. Because the time to recovery of gastrointestinal function cannot be converted into a binary outcome (response or non-response), we did not include this study when performing the meta-analysis on the response rate. The pooled analysis showed a significant clinical benefit of acupuncture combined with conventional treatment over conventional treatment alone in improving the overall response rate [RD = 0.16, 95% CI (0.11, 0.21), *p <* 0.001], with no heterogeneity across studies (*I*^2^ = 0%, *p =* 0.99) (Figure 3).

#### 3.4.2 The effectiveness of acupuncture on gastric draining amount

**Figure 4:**
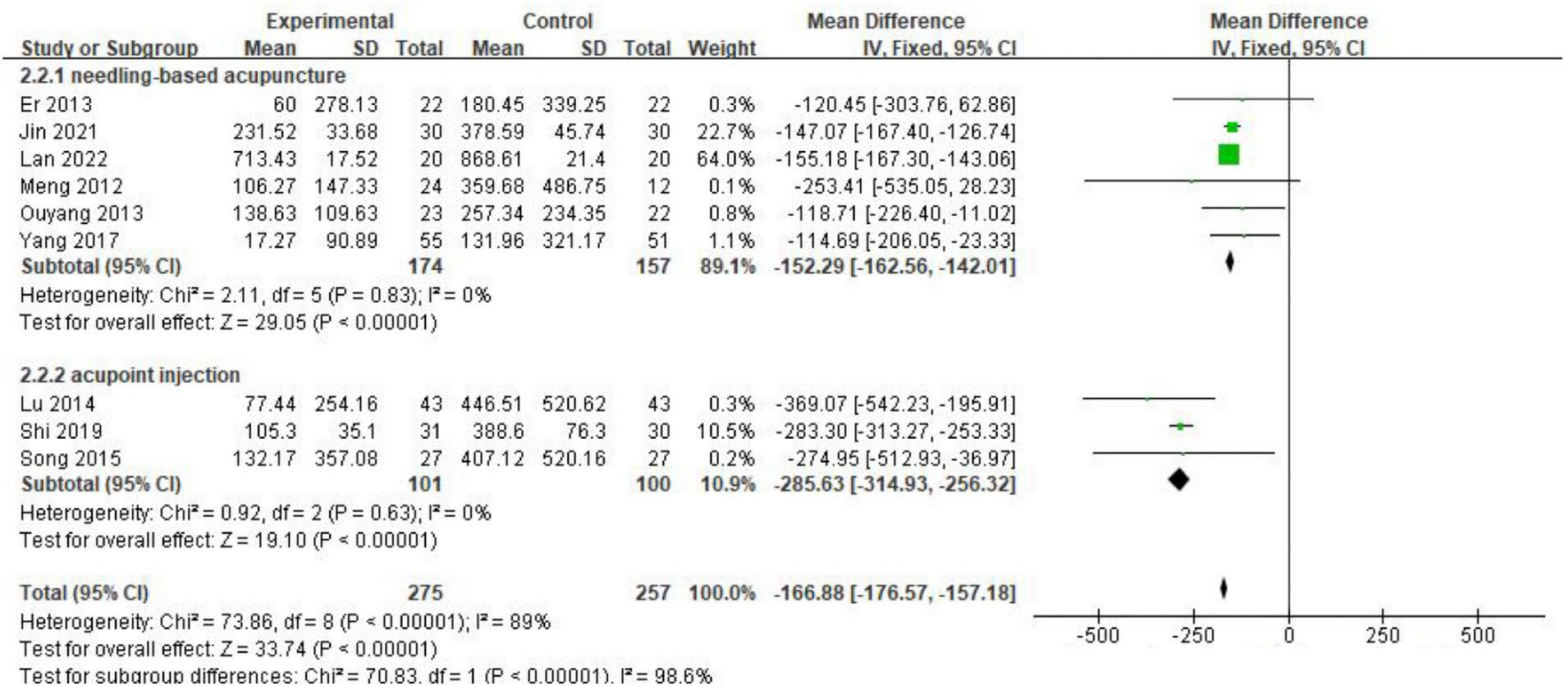
Forest plot of the decrease of gastric drainage after treatment. The coordinate values corresponding to the green squares represent the additional reduction in gastric drainage volume brought about by acupuncture in each study. The size of the square represents the weight of each study. The range of values spanned by the horizontal line represents the confidence interval. The top and middle diamonds represent the results of the subgroup analysis. The diamond at the bottom represents the overall effect.

Nine studies (13–17, 19, 21, 23, 24) with 532 patients reported the amount of gastric juice drainage. The meta-analysis revealed that acupuncture significantly reduced gastric drainage [MD = −166.88, 95% CI (−176.57, −156.18), *p <* 0.001], but significant heterogeneity was observed between studies (*I*^2^ = 89%, *p <* 0.001). Subgroup analysis identified the form of acupuncture as the source of this heterogeneity. The studies were divided into two groups: those using acupoint injection and those using traditional needle-based acupuncture. After grouping, no heterogeneity was found within either group (*I*^2^ = 0%, *p =* 0.83 for acupoint injection; *I*^2^ = 0%, *p* = 0.63 for traditional acupuncture). A fixed-effects model was used for the meta-analysis. The results indicated that acupuncture combined with conventional treatment was more effective than conventional treatment alone in reducing gastric juice drainage. Studies using acupoint injection showed a larger reduction [MD = −285.63, 95% CI (−314.93, −256.32), *p <* 0.001] compared to those using traditional needle-based acupuncture [MD = −152.29, 95% CI (−162.56, −142.01), *p <* 0.001] (Figure 4).

#### 3.4.3 The effectiveness of acupuncture on gastric tube indwelling time

**Figure 5:**
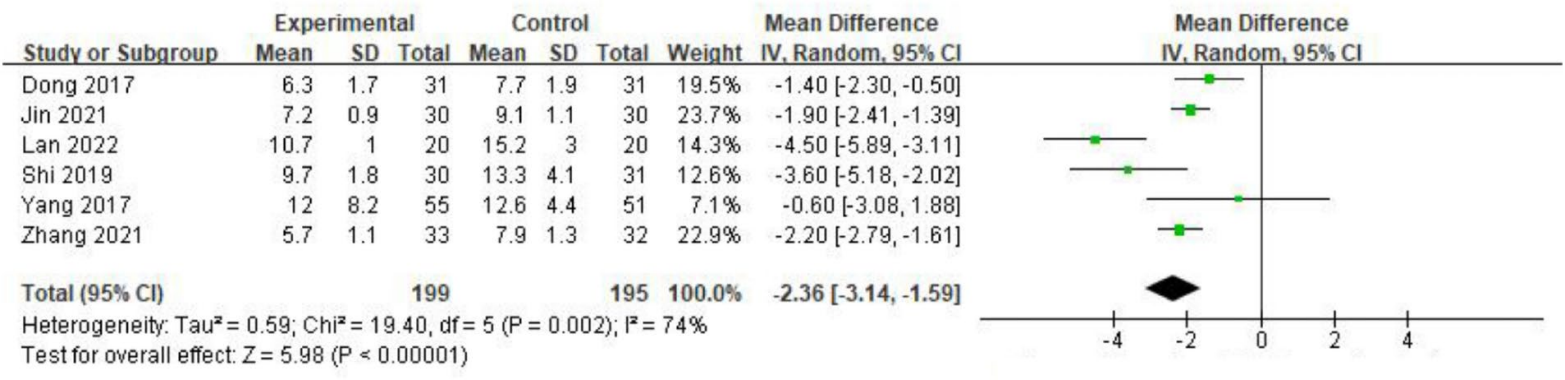
Forest plot of the decrease of gastric tube indwelling time after treatment. The coordinate values corresponding to the green squares represent the additional reduction in gastric tube removal time brought about by acupuncture in each study. The size of the squares represents the weight of each study. The range of values spanned by the horizontal line represents the confidence interval. The diamond at the bottom represents the combined result.

Six studies (13, 14, 16, 18, 19, 21) involving 394 patients reported the effect of acupuncture on gastric tube indwelling time. The heterogeneity test indicated moderate variability (*I*^2^ = 74%, *p =* 0.002), prompting the use of a random-effects model. The meta-analysis results showed that acupuncture combined with conventional treatment significantly shortened gastric tube indwelling time compared to conventional treatment alone [MD = −2.36, 95% CI (−3.14, −1.59), *p <* 0.001] (Figure 5).

#### 3.4.4 The effectiveness of acupuncture on serum motilin level

**Figure 6:**
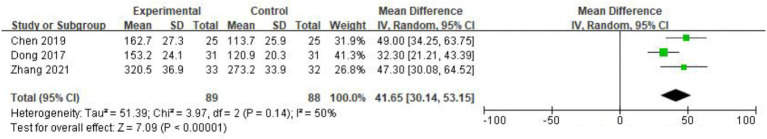
Forest plot of the increase of serum motilin level after treatment. The coordinate values corresponding to the green squares represent the additional increase in serum motilin brought about by acupuncture in each study. The size of the squares represents the weight of each study. The numerical range spanned by the horizontal line represents the confidence interval, and the diamond at the bottom represents the combined result.

Three studies (16, 18, 20) reported changes in serum motilin levels before and after treatment, involving 177 patients. Moderate heterogeneity was found (*I*^2^ = 74%, *p =* 0.002), so a random-effects model was applied. The pooled results showed that acupuncture significantly increased serum motilin levels, thereby improving gastrointestinal motility [MD = 41.65, 95% CI (30.14, 53.15), *p <* 0.001] (Figure 6).

### 3.5 Safety analysis

Four studies (14, 21, 23, 24) have reported the safety data and found there was no adverse event in both acupuncture and control groups. However, the remaining studies did not record or report safety data in detail. Based on the available data, acupuncture treatment appears to be safe for patients with PGS. However, due to the lack of comprehensive safety data, we were unable to conduct a systematic evaluation of the safety of acupuncture, suggesting that acupuncture may be safe. However, due to the lack of comprehensive safety evaluations, it is not possible to draw definitive conclusions. Further monitoring of adverse events is necessary to clarify the clinical safety of acupuncture.

### 3.6 Publication bias

**Figure 7:**
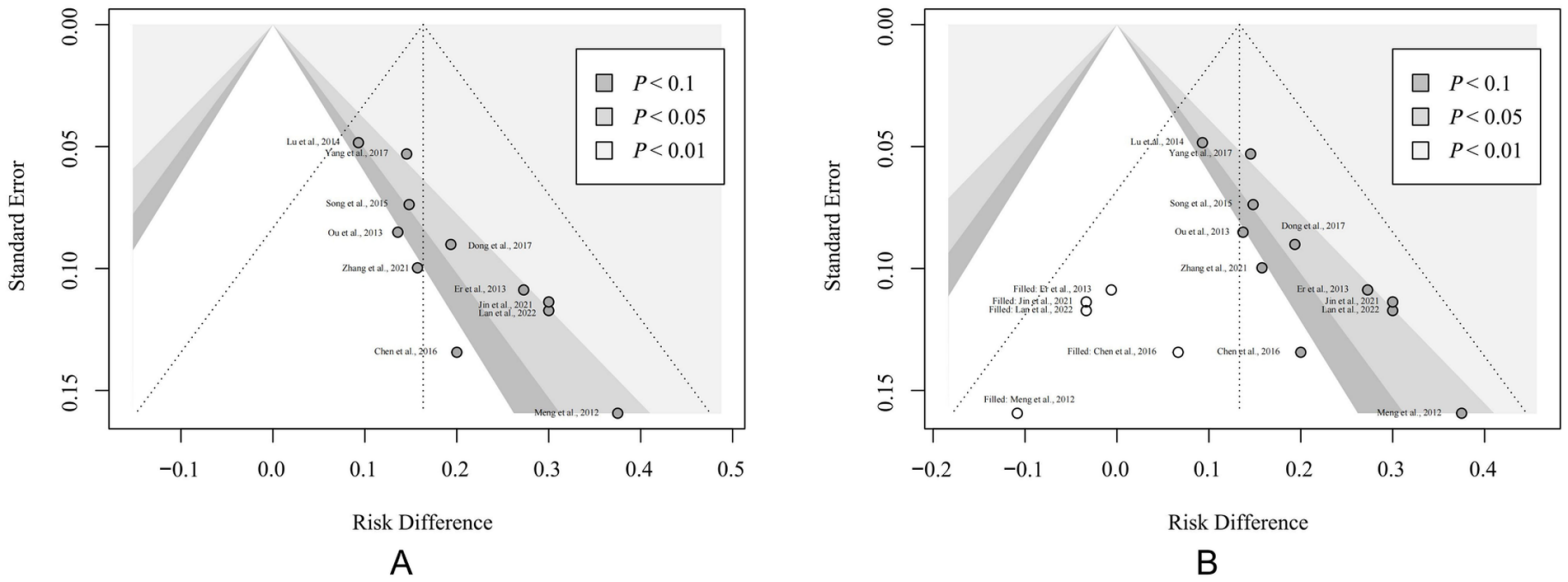
Results of sensitivity analysis. **(A)** Funnel plot of the overall response rate. **(B)** Funnel plot after the correction of trimming and filling method. The solid circles represent each included study. The hollow circles represent virtual studies added based on the trimming and filling method.

Publication bias was assessed using funnel plots and Egger’s test. The analysis of the overall response rate, which was reported in more than 10 studies, indicated potential publication bias. The funnel plot for this outcome was asymmetrical (Figure 7A), and Egger’s test result (*p =* 0.006) further supported this finding. After adjusting for publication bias using the trimming and filling method, the significant advantage of acupuncture in improving the overall response rate remained [RD = 0.13, 95% CI (0.09, 0.18), *p <* 0.001 (Figure 7B)]. Potential sources of bias include geographic limitations, variability in intervention formats, and the absence of negative results.

## 4 Discussion

### 4.1 Summary of evidence

We conducted a meta-analysis to comprehensively assess the role of acupuncture in improving PGS. From the published literature, we selected 12 studies involving 709 patients. Our analysis included all needle-based acupuncture forms, including manual acupuncture, electroacupuncture, and acupoint injection. The results demonstrated that acupuncture significantly alleviated clinical symptoms of PGS and enhanced the overall treatment response. In addition to the beneficial effects on subjective symptoms, acupuncture improved objective indicators, such as gastric tube indwelling time, gastric juice volume, and serum motilin levels. These findings suggest that acupuncture effectively enhances gastrointestinal motility, alleviates digestive discomfort, and shortens the duration of PGS. Subgroup analysis indicated that acupoint injection might yield more significant effects compared to conventional acupuncture; however, this result may be influenced by random errors due to limited follow-up, small sample sizes, or the absence of a sham acupuncture control. Additionally, due to insufficient safety information, the assessment of acupuncture’s clinical efficacy in this study may be overstated, potentially failing to adequately account for the associated risks. Consequently, while our review concluded that acupuncture is effective in treating PGS, it is crucial to approach this conclusion with significant caution.

### 4.2 Advantages and limitations

Acupuncture is a non-pharmacological treatment that can promote gastrointestinal function without the risk of interaction with conventional therapies, making it a promising option in clinical practice. However, it is important to consider that the efficacy of acupuncture in treating functional gastrointestinal disorders may be influenced by the placebo effect. Researchers found that sham acupuncture (needling non-acupoint areas) can also alleviate patients’ digestive discomfort, which suggests that we need to use more objective indices to evaluate efficacy (25). However, another study found that compared to sham acupuncture, acupuncture at acupoints can significantly increase serum brain-gut peptides (such as motilin and gastrin) levels, not just subjective satisfaction, and lead to more significant and stable therapeutic effects (26). To address these concerns, our review included objective indicators and found that acupuncture not only relieves subjective symptoms but also significantly reduces gastric juice volume, shortens gastric tube retention time, and increases serum motilin levels, thus supporting its role in promoting gastrointestinal motility recovery. In addition to the inclusion of objective indicators, we also applied appropriate statistical methods to address the issues in this meta-analysis that may bias the results. Sensitivity analysis suggested that efficient pooled results may be affected by the risk of bias. To mitigate this, we used the trimming and filling method to correct for such bias. In dealing with heterogeneity, we used subgroup analysis to further explore the reasons for heterogeneity and discover the potential advantages of acupoint injection therapy. In summary, the rigorous methodology and the inclusion of objective indicators strengthen the objectivity and persuasiveness of our conclusions.

Our review has several limitations. Despite using a rigorous methodology, the included studies have certain shortcomings. First, since “Deqi” (the sensation of needle sensation) is a key component in clinical acupuncture studies and is dependent on the practitioner’s technique, it is challenging to conduct a fully blinded, double-blind study. However, future research could adopt blinded evaluation methods to reduce potential bias. Second, we included studies that utilized random number tables for randomization, which, while commonly used, have certain systematic flaws. These tables are pre-set before the experiment and will potentially introduce artificial bias. Additionally, this method cannot accommodate stratified sampling, which could lead to confounding factors such as age and gender affecting the results. Finally, many included trials did not adequately report the safety information of acupuncture, which was a significant limitation. PGS patients in the recovery period after surgery may have a relatively fragile immune system. Although acupuncture is generally considered a low-risk treatment method, any intervention may cause local reactions or other adverse reactions, especially infection at the acupuncture site, needle dizziness, etc. Failure to fully evaluate safety may affect the reliability of our conclusion. Therefore, in clinical practice, medical staff should still use acupuncture treatment for PGS patients with caution, especially for patients with bleeding tendencies, immune system diseases, or other contraindications to acupuncture.

### 4.3 Comparison with previous studies

There were several previous systematic reviews that can be compared with our review. Cheong et al. (10) reported that acupuncture significantly improves clinical symptoms, either as a monotherapy or as an adjunct to other treatments. However, this review only reported response rates as the primary outcome, and it included studies where acupuncture was combined with oral herbal decoctions, which could have interfered with the precision of the results. Another meta-analysis focused on acupuncture for symptomatic gastroparesis, but it primarily concentrated on diabetic gastroparesis, including only one study on PGS (27). In contrast, our review focused specifically on PGS and incorporated objective measures, such as gastric drainage, gastric tube indwelling time, and serum motilin levels. By limiting the intervention to needle-based acupuncture techniques, we aimed to provide more accurate and reliable data.

### 4.4 Implications for research

Acupuncture, recognized as a globally practiced complementary therapy, has been extensively utilized in the treatment of functional gastrointestinal disorders such as functional dyspepsia (28), irritable bowel syndrome (29), and gastroparesis (30). Our review suggests that acupuncture may aid in gastrointestinal recovery and provide symptomatic relief for patients with PGS. However, the safety of acupuncture in this patient population remains uncertain, as most studies fail to adequately report safety data. This significant lack of safety information raises concerns about the reliability of the findings and their broader applicability. In addition, understanding the placebo effect is crucial, as patient expectations may influence perceived efficacy, masking true treatment effects (25). To strengthen internal validity, future research should consider incorporating more rigorous methods, along with comprehensive safety reporting to clarify the potential risks of acupuncture. Given these limitations, the findings of this review should be interpreted with caution.

It is imperative to further investigate the mechanism of action underlying acupuncture, as this knowledge could significantly enhance its clinical efficacy. Previous studies have indicated that the effects of acupuncture on gastrointestinal motility may be intricately linked to the activation of the vagus nerve (31), gastrointestinal hormone levels (32), intestinal flora (33), etc. Our study demonstrated that acupuncture treatment can significantly elevate serum motilin levels. Motilin, secreted by the enterochromaffin cells in the duodenal and jejunal mucosa, plays a crucial role in stimulating gastrointestinal motility. These findings suggest that the enhancement of gastrointestinal motility through the modulation of brain-gut peptides may underlie the mechanism of acupuncture. However, the precise mechanism by which acupuncture influences duodenal mucosal epithelial cells requires further elucidation in future research.

## 5 Conclusion

This meta-analysis indicates that acupuncture may offer a beneficial effect in treating PGS, improving clinical symptoms, gastrointestinal motility, and serum motilin levels. However, the lack of safety data in the included studies limits the reliability and generalizability of research conclusions. Therefore, well-designed RCTs with more rigorous randomization, sham acupuncture controls, and comprehensive safety reporting are necessary to refine these results. At present, we must view these findings with caution and strictly follow the contraindications of acupuncture treatment in clinical practice.

## Data Availability

The original contributions presented in the study are included in the article/Supplementary material, further inquiries can be directed to the corresponding author.

## Footnotes

^1^ www.inplasy.com
