# Supplementary material for: Efficacy and safety of acupuncture for postoperative gastroparesis syndrome: a systematic review and meta-analysis
Source: Front Med (Lausanne). 2025 Jan 6;11:1494693. doi: 10.3389/fmed.2024.1494693 (PMC11743162; doi:10.3389/fmed.2024.1494693)
Supplement: Supplementary file 1 [file Table_1.DOCX]

**Supplementary Table 1 Literature search strategy for PubMed**

| Search Strategy (PubMed) | |
| --- | --- |
| #1 | Acupuncture [Mesh] |
| #2 | Acupoint Injection[Title/Abstract] |
| #3 | Warm Acupuncture [Title/Abstract] |
| #4 | Electroacupuncture [Title/Abstract] |
| #5 | Pharmacopuncture [Title/Abstract] |
| #6 | #1 or #2 or #3 or #4 or #5 |
| #7 | Gastroparesis [Mesh] |
| #8 | Gastric Stasis [Title/Abstract] |
| #9 | Gastric Stases [Title/Abstract] |
| #10 | Stases, Gastric [Title/Abstract] |
| #11 | Stasis, Gastric [Title/Abstract] |
| #12 | #7 or #8 or #9 or #10 or #11 |
| #13 | #6 and #12 |
